# The status of job burnout and its influence on the working ability of copper-nickel miners in Xinjiang, China

**DOI:** 10.1186/s12889-020-8245-4

**Published:** 2020-03-06

**Authors:** Xuemei Sun, Li Zhang, Chen Zhang, Jiwen Liu, Hua Ge

**Affiliations:** 1 Department of Public Health, Xinjiang Medical University, Urumqi, 830011 China; 2 Human Resource Department, The First Affiliated Hospital of Xinjiang Medical University, Urumqi, 830011 China

**Keywords:** Miners, Job burnout, Working ability

## Abstract

**Background:**

Job burnout is increasingly common among occupational groups, and it is evolving into a new occupationally harmful phenomenon. The purpose of this study was to investigate the status of job burnout and its influence on the working ability of copper-nickel miners in Xinjiang, China, and to provide a theoretical basis for alleviating job burnout and improving the working ability of copper and nickel miners.

**Methods:**

A cross-sectional survey was carried out in June 2017 to September 2018 in Hami City, Xinjiang Autonomous Uygur Region, China. According to the main production process stratification of copper-nickel ore (mining unit, beneficiation unit, smelting unit), a self-administered questionnaire survey on the general situation of miners was conducted on the basis of the Chinese Maslach Burnout Inventory and Work Ability Index Questionnaire among 1400 miners registered in the human resources department of copper and nickel mines by stratified cluster sampling.

**Results:**

There were 1014 miners with different degrees of burnout, accounting for 80.86% of the total: 432 people reported mild burnout (34.45%), 516 reported moderate burnout (41.15%), and 66 reported high burnout (5.26%). There were significant differences in the degree of burnout according to sex, age, education level, monthly income, and work unit (*p* < 0.05). The level of male burnout was higher than that of females. Miners aged 35–40 years, with a high-school education, a monthly income of less than 2500 yuan, and who belonged to the smelting unit had the highest job burnout.There were significant differences in working ability among miners with different burnout level (*p <* 0.01). Partial correlation analysis showed that work ability was negatively correlated with emotional exhaustion, reduced sense of achievement, and total burnout score (*p* < 0.001). Multinomial logistic regression analyses showed that the education level, professional title,work units and job burnout level had a pronounced impact on the working ability of miners (*p* < 0.001); The reduced level of education, primary title, smelting unit, and the increase in job burnout are risk factors for the reduction of working ability.

**Conclusions:**

Our results indicate that job burnout is common among copper and nickel miners. Furthermore, working ability decreases with an increase in job burnout, and reducing job burnout can improve the working ability of copper and nickel miners.

## Background

Burnout refers to the state of emotional, mental and physical exhaustion caused by persistent stress at work [1], including emotional exhaustion, depersonalization, and reduced personal accomplishment [2]. With rapid social development, production technology is changing. Work is increasingly complex and diverse, the pace of work is accelerating, and social competition is becoming increasingly fierce. People feel that pressures from work have significantly increased, and a sense of job burnout among workers is increasingly common. Job burnout is evolving into a new occupationally harmful phenomenon that has a wide impact on health, and researchers in occupational therapy and medicine are paying more attention to it. Job burnout has become an important indicator reflecting the mental health of the occupational population. The impact of job burnout on health has become a new challenge for the global occupational health community [3].

There are many factors that lead to job burnout. In general, they can be divided into four categories. The first category relates to organizational management. Organizational fairness, support, climate, and situation, along with the leadership style, the reward and punishment system, and the incentive mechanism are all related to the occurrence of job burnout [4]. Studies have shown that manpower allocation, compensation and benefits, the working environment, the current management level, and competence for jobs are factors that are related to job burnout [5]. There is also research suggesting that when employees’ legal rights are not guaranteed and when they are not respected, it can drastically dampen employees’ professional feelings and induce the occurrence of burnout [6]. The second category pertains to occupational factors. Studies have pointed out that the occurrence of job burnout is closely related to such factors as work pressure, workload, working hours, and work content [7–9]. In addition, research has uncovered a correlation between job satisfaction and job burnout: a few dimensions of job satisfaction can significantly contribute to job burnout, and individual job dissatisfaction is an important cause of job burnout [10]. The third category encompasses social support factors. Social support is an important concept related to job burnout [11]. One study showed that social support from family members, friends, colleagues, and leaders is of great significance in reducing the level of employee burnout, and is beneficial to the individual’s physiological and psychological response [12]. The final category comprises individual factors. One study pointed out that factors such as personality, gender, age, marital status, and education affect the occurrence of burnout [13]. In addition, personal lifestyle [14], smoking, excessive drinking [15], lack of exercise, and obesity [16] are associated with the occurrence of burnout.

The harm from job burnout is multifaceted. It not only affects the physical and mental health of the worker, it also affects the work itself and the organization to which the worker belongs. First, long-term burnout can damage the physical and mental health of workers [17]. In terms of mental health, burnout can cause or induce depression and anxiety [18]. In terms of physiology, job burnout leads to a greater risk of chronic fatigue, headaches, hypertension, cardiovascular disease, type II diabetes, gastrointestinal disturbances, and sleep disorders [19]. Studies have shown that burnout may lead to lower job satisfaction, the interruption of interpersonal relationships, drug abuse, depression, and even suicide [20, 21]. Second, job burnout can reduce employee work efficiency, increase the absence rate, and even lead to a higher turnover rate [22]. By studying the effects of job burnout on interpersonal relationships, efficiency, and organization, scholars have found that job burnout has a significantly negative effect on individual job performance, and that it can cause anxiety and dissatisfaction among employees and aggravate their retreat from work. Typical manifestations are indifference to colleagues and irresponsibility for work. This not only reduces individual productivity and self-efficacy, it also degrades individual commitment and loyalty to the organization, which can lead to a loss of confidence in work and life and even induce depression. Moreover, these sentiments can be spread to others, affecting the overall morale of the organization and general efficiency [23, 24]. Third, burnout has brought economic burdens to countries and enterprises [25]. The physical and mental discomfort experienced by employees and the poor performance resulting from job burnout impose additional financial burdens on enterprises. According to one study, 26% of workers in the United States suffer from job burnout or excessive stress, and the annual medical burden for this problem is US$190 billion [26]. The negative impact from job burnout is also of considerable interest in the field of occupational psychology. Indeed, preventing and controlling job burnout is crucial to improving the physical and mental health of the occupational population, and forms part of the basis for ensuring good organization and sustainable and efficient social and economic development.

The impact of job burnout on work efficiency and ability has been gradually acknowledged by scholars. Working ability is an overall manifestation of a worker’s capacity to solve and cope with labor tasks during the working process. The demand from labor tasks on workers is multifaceted, including physical demands, mental demands, and social demands. To meet these needs successfully, workers must have the corresponding physical, mental and social abilities. The working ability is also closely related to workers’ professional experience, psychological state, work motivation, and so on [27]. Many studies have confirmed the impact of job burnout on work ability. Job burnout can negatively affect workers’ emotions, cognition, and behavior, and it can harm their physical and mental health, work status, and quality of life, thereby reducing their work ability [28–30].

Copper and nickel miners belong to a special occupational group. Their working environment is rather harsh. They carry out repetitious tasks, with a heavy work burden, high labor intensity, long working hours, inadequate rest, and exposure to toxic and harmful substances. Further, they are affected by many sources of work pressure in the production process. Often, they work at sites that are relatively remote, far from family and friends, and their interpersonal environment is relatively simple, with less social support. Working life is relatively monotonous, with a lack of recreational facilities, and the social status and salary of miners are low. This combination of factors makes miners prone to burnout, which can affect their physical and mental health and working ability [31]. In recent years, scholars have paid more attention to the phenomenon of job burnout in different occupational groups. Previous studies on job burnout mostly focus on teachers, medical staff, police, civil servants, and other occupational groups [32, 33]. There is relatively little research on the special occupational group of copper and nickel miners. In this study, a self-assessment questionnaire was administered to copper and nickel miners in Xinjiang to investigate the status of job burnout and its influence on the working ability of copper-nickel miners in Xinjiang, China, and to provide a theoretical basis for alleviating job burnout and improving the working ability of copper and nickel miners.

## Methods

### Study population

From June 2017 to September 2018, in accordance with the main production processes of a copper-nickel mine (mining unit, beneficiation unit, smelting unit), this study used stratified cluster sampling to administer a self-assessment questionnaire to all registered workers who have been on the job for more than one year, with information provided by the Human Resources Department of a copper-nickel mine in Hami City, Xinjiang Autonomous Uygur Region, China. Inclusion criteria: The subjects were miners aged 18 to 60 who worked for more than one year, volunteered to participate, and filled in an informed consent form. Exclusion criteria: Miners with severe cognitive impairment or a previous history of dementia or psychosis were excluded. The investigation was approved by the Ethics Committee of the First Affiliated Hospital at Xinjiang Medical University (Approval Number: 20170214–174). A total of 1400 questionnaires were sent out and 1254 valid questionnaires were collected; the validity rate was thus 89.57%.

### Research methods

A questionnaire (detailed below) was used to investigate the status of job burnout and its impact on working ability.

#### General investigation

This section discusses general demographic characteristics such as sex, age, number of working years, educational level, marital status, professional title, monthly income, and work units.

#### Job burnout investigation

Job burnout as experienced by the subjects was evaluated using the Chinese Maslach Burnout Inventory (CMBI), which is a Chinese-version of the questionnaire prepared by Li Yongxin [34, 35]. There are 15 items in the questionnaire, with three dimensions and five items in each dimension. They are specifically distributed under emotional exhaustion, depersonalization, and reduced personal accomplishment. The questionnaires are scored with seven grades: “1” denotes total inconsistency, “7” denotes complete consistency, and from “1” to “7” represents a low to high degree of compliance. Among the dimensions, reduced personal accomplishment uses reverse scoring, and each dimension is scored between 5 and 35. The higher the score in the three dimensions, the more pronounced the burnout. According to the critical values of the three dimensions (emotional exhaustion score = 25, depersonalization score = 11, reduced personal accomplishment score = 16), job burnout was divided into four levels: zero burnout (score of three dimensions < critical value), mild burnout (score of one dimension ≥ critical value), moderate burnout (score of two dimensions ≥ critical value), and high burnout (score of three dimensions ≥ critical value). The CMBI evaluation results for occupational groups showed that the scale was reliable and valid [36]. At present, the questionnaire has been widely used in teachers, medical staff, police, corporate managers and employees, marketers, librarians and other professional fields.

#### Working ability investigation

The Work Ability Index Questionnaire (WAI) was used to evaluate working ability. This was developed in 1994 by the Finnish National Institute of Occupational Health after years of research and development. Laiji Ma is equivalent to the introduction of the WAI scale in 1994 and translated into Chinese. It has been used in professional fields, and its reliability and validity have been tested and evaluated extensively. It is a test tool used to judge whether a person can finish his/her work continuously. At present, the scale has been widely used in occupational fields such as medical personnel, teachers, drivers, oil workers, and miners. It includes seven items: self-evaluation of work ability; adaptability of physical condition; condition of illness and injury; influence on work; days absent from work; prediction of working ability after two years; and mental health status(are you happy with your daily activities, have you been active and focused lately, are you hopeful about the future recently). The total score of the WAI is calculated by summing the scores of all items, which range from 7 to 49 points. The higher the score, the better the ability to work. Working ability is divided into four levels according to the WAI score: 7–27 points (ability to work is poor), 28–36 points (ability to work is moderate), 37 to 43 points (ability to work is good), and 44 to 49 points (ability to work is excellent) [37].

#### Quality control

Before the formal investigation, we contacted the subjects under investigation to secure their active cooperation and conduct a small-scale pre-investigation. We set up a professional investigation team. The investigators had relevant medical knowledge and professional psychological experience. Investigators collected the contents of the questionnaire, and determined the terminology and methods of investigation. To improve responsiveness, small souvenirs were given to the subjects. The questionnaires were reviewed centrally by the investigators upon receipt and collected immediately upon completion. They were then numbered and reviewed uniformly, and questionnaires that were less than 80% complete were eliminated.

#### Statistical methods

Statistical analysis was performed with SPSS 21.0 (SPSS Inc., Chicago, IL, USA). A normality test of the measurement data was carried out. Data that did not conform to a normal distribution were described by the median (*M*) and quartile (*Q*). The Mann–Whitney *U* test was used to compare two groups and the Kruskal–Wallis *H* test was used to compare more groups of nonparametric (non-normally distributed) data. Partial correlation was used to analyze the correlation of variables. Multinomial logistic regression was employed for multivariate analysis. The significance level (α) was set at 0.05.

## Results

### General demographic characteristics of copper-nickel miners

Among the 1254 copper-nickel miners, 1116 were men (89.00%) and 138 were women (11.00%). The average age was 32.31 ± 9.09 years (Table 1).

**Table 1:** Characteristics of the copper-nickel worker sample population

Items	Groups	Case Number	Percentage (%)
Sex	Male	1116	89.00
	Female	138	11.00
Age (years)	< 25	327	26.08
	25~	339	27.03
	30~	159	12.68
	35~	132	10.53
	40~	174	13.88
	45~	123	9.81
Number of working years	≤5	666	53.11
	6~	450	35.89
	11~	138	11.00
Education level	Junior high school and below	204	16.27
	High school	297	23.68
	Junior college	627	50.00
	Bachelor’s degree or above	126	10.05
Marital status	Unmarried	444	35.41
	Married	810	64.59
Professional title	None	813	64.83
	Primary	216	17.22
	Intermediate and above	225	17.94
Income (yuan)	< 2500	252	20.10
	2500~	582	46.41
	3000~	171	13.64
	3500~	108	8.61
	4000~	141	11.24
work units	Mining unit	441	35.17
	Beneficiation unit	267	21.29
	Smelting unit	546	43.54
Total		1254	100%

### Detection rate of job burnout

The results showed that there were 1014 miners with varying degrees of job burnout, accounting for 80.86% of the total. Among these 1014 miners, 432 (34.45%) were associated with mild burnout, 516 (41.15%) with moderate burnout, and 66 (5.26%) with high burnout (Table 2).

**Table 2:** Detection Rate of Job Burnout

Burnout level	Case Number	Percentage (%)
Zero burnout	240	19.14
Mild burnout	432	34.45
Moderate burnout	516	41.15
High burnout	66	5.26
Total	1254	100%

### Comparison of job burnout levels in different populations

There were significant differences in depersonalization and total burnout scores between the sexes (*p* < 0.05), and the level of male burnout was higher than that of females. There were significant differences in emotional exhaustion, reduced sense of achievement, and total burnout scores among different age groups (*p* < 0.001), and the highest level of burnout was found among those aged 35–40 years. There were also significant differences among different education levels, monthly income, and work units groups in all dimensions (*p* < 0.001). Those who had not been educated beyond junior high school scored the highest in reduced personal accomplishment. Miners with a high-school education, a monthly income of less than 2500 yuan, and who belonged to the smelting unit had the highest job burnout (Table 3).

**Table 3:** Comparison of job burnout levels in different populations

Groups	Case Number	Job burnout			
		emotional exhaustion[M(Q)]	depersonalization[M(Q)]	reduced personal accomplishment[M(Q)]	Total burnout score[M(Q)]
Sex					
Male	1116	18.0(12.0)	13.0(11.0)	16.0(7.0)	49.0(21.0)
Female	138	16.0(13.0)	11.0(11.0)	15.0(10.0)	45.0(21.0)
*Z*-Value		−1.09	−3.09	−0.90	−2.11
*p*-Value		0.28	<0.01	0.37	0.04
Age (years)					
< 25	327	18.0(11.0)	12.0(11.0)	16.0(7.0)	47.0(22.0)
25~	339	18.0(11.0)	14.0(13.0)	17.0(8.0)	50.0(19.0)
30~	159	20.0(12.0)	14.0(11.0)	16.0(8.0)	51.0(21.0)
35~	132	18.5(11.0)	11.0(10.0)	15.5(6.5)	48.5(22.5)
40~	174	17.5(10.0)	13.0(10.0)	19.0(8.0)	48.0(20.0)
45~	123	14.0(11.0)	10.0(10.0)	15.0(12)	44.0(22.0)
Chi-Squared Value		21.49	7.83	18.44	18.15
*p*-Value		<0.01	0.17	<0.01	<0.01
Number of working years					
≤ 5	666	19(11.0)	13(12.0)	16(7.0)	49(21.0)
6~	450	18(11.0)	13(10.0)	18(9.0)	49(21.0)
11~	138	18(12.0)	11(11.0)	13(8.0)	45(22.0)
Chi-Squared Value		3.55	0.96	46.38	4.50
*p*-Value		0.17	0.62	<0.01	0.11
Education level					
Junior high school and below	204	13.0(13.0)	10.5(8.0)	19.5(11.5)	48.0(17.0)
High school	297	19..0(11.0)	14.0(12.0)	17.0(7.0)	52.0(16.0)
Junior college	627	19.0(10.0)	13.0(11.0)	16.0(8.0)	49.0(22.0)
Bachelor’s degree or above	126	14..0(10.0)	11.0(9.0)	14.0(10.0)	42.5(19.0)
Chi-Squared Value		36.97	21.38	63.00	34.76
*p*-Value		<0.01	<0.01	<0.01	<0.01
Marital status					
Unmarried	444	18.0(11.0)	12.0(11.0)	16.0(7.0)	47.0(21.0)
Married	810	18.0(12.0)	13.0(11.0)	16.0(9.0)	49.0(22.0)
*Z*-Value		−1.46	−0.27	−0.28	−1.07
*p*-Value		0.14	0.79	0.78	0.28
Professional title					
None	813	18.0(12.0)	13.0(11.0)	17.0(8.0)	49.0(21.0)
Primary	216	18.0(11.0)	11(10.5)	16(7.5)	47.5(19.0)
Intermediate and above	225	19.0(9.0)	13.0(11.0)	15.0(10.0)	47.0(24.0)
Chi-Squared Value		0.54	1.37	11.82	5.47
*p*-Value		0.76	0.50	<0.01	0.07
Income (yuan)					
< 2500	252	19.0(10.0)	13.0(12.0)	17.0(7.0)	52.0(22.0)
2500~	582	19.0(12.0)	14.0(12.0)	16.0(7.0)	50.0(21.0)
3000~	171	13.0(10.0)	9.0(8.0)	20.0(13.0)	46.0(14.0)
3500~	108	18.0(12.0)	12.0(9.0)	18.0(8.5)	49.5(21.0)
4000~	141	15.0(13.0)	9.0(8.0)	13.0(7.0)	39.0(16.0)
Chi-Squared Value		33.21	52.43	86.35	53.68
*p*-Value		<0.01	<0.01	<0.01	<0.01
Work units					
Mining unit	441	16.0(12.0)	10.0(9.0)	16.0(10.0)	46.0(20.0)
Beneficiation unit	267	19.0(10.0)	12.0(11.0)	15.0(6.0)	46.0(23.0)
Smelting unit	546	19.0(11.0)	14.0(11.0)	18.0(6.0)	52.0(17.0)
Chi-Squared Value		22.65	47.29	22.32	41.08
*p*-Value		<0.01	<0.01	<0.01	<0.01

### Comparison of working ability scores at different burnout level

There were significant differences in working ability among miners with different burnout level (*p <* 0.001). With an increase in burnout level, the scores decreased for working ability. This suggests that the higher the burnout level of copper and nickel miners, the lower their working ability (Table 4).

**Table 4:** Comparison of WAI scores at different burnout level

Burnout level	Case Number	WAI score[M(Q)
Zero burnout	240	48.0(13.0)
Mild burnout	432	45.0(13.0)
Moderate burnout	516	44.0(11.0)
High burnout	66	41.0(9.0)
Chi-Squared Value		102.39
*p*-Value		< 0.001

### Relevance analysis of job burnout and working ability

After controlling for confusing general demographic data, partial correlation analysis showed that work ability was negatively correlated with emotional exhaustion, a reduced sense of achievement, and total burnout score (*p* < 0.001). This suggests that the more intense the job burnout is, the lower the job ability (Table 5).

**Table 5:** Relevance analysis of job burnout and working ability

Factors	*r*-Value	*p*-Value
emotional exhaustion	−0. 132	< 0.001
depersonalization	− 0. 046	0. 101
reduced personal accomplishment	−0. 200	< 0.001
Total burnout score	−0. 194	< 0.001

### Exploration of factors influencing working ability

Multinomial logistic regression was used to analyze the effects of the different characteristics of the sample population as well as the way in which job burnout experienced by copper-nickel miners influences working ability. Taking working ability as the dependent variable, after adjusting for general demographic factors such as sex, age, number of working years, education level, marital status, professional title, income, work units, the results of multinomial logistic regression analysis showed: education level, professional title, work units and job burnout level have a comprehensive impact on working ability. The reduced level of education, primary title, smelting unit, and the increase in job burnout are risk factors for the reduction of working ability. The result suggests that the higher the level of job burnout, the lower the working ability (*p* < 0.05) (Table 6 and Table 7).

**Table 6:** Assignment of factor-specific variables

Variable	Name	Assignment
y	WAI	1 = medium, 2 = good, 3 = excellent
×1	Sex	0 = male, 1 = female
×2	Age	Accurate values
×3	Number of working years	Accurate values
×4	Education level	0 = bachelor’s degree or above, 1 = Junior college, 2 = High school, 3 = Junior high school and below
×5	Marital status	0 = Unmarried, 1 = Married
×6	Professional title	0 = Intermediate and above, 1 = Primary, 2 = None
×7	Income	Accurate values
×8	Work units	0 = Mining unit, 1 = Beneficiation unit, 2 = Smelting unit
×9	Job burnout	0 = Zero burnout, 1 = Mild burnout, 2 = Moderate burnout, 3 = High burnout

**Table 7:** Effects of quality-of-life-related factors among copper-nickel miners according to the results of multinomial logistic regression analyses

Factor	Medium					Good				
	*Exp(B)*	*S.E.*	*Wald*	*p*-Value	*OR*(*95%CI*)	*Exp(B)*	*S.E.*	*Wald*	*p*-Value	*OR*(*95%CI*)
Education level										
Junior high school and below	–	–	–	–	1	–	–	–	–	1
High school	−1.120	0.313	12.796	< 0.001	0.33(0.18,0.60)	0.013	0.220	0.004	0.952	1.01(0.66,1.56)
Junior college	−1.943	0.325	35.800	< 0.001	0.14(0.08,0.27)	−0.624	0.215	8.417	0.004	0.54(0.35,0.82)
bachelor’s degree or above	−2.597	0.679	14.622	< 0.001	0.07(0.02,0.28)	−0.228	0.285	0.642	0.423	0.80(0.46,1.39)
Professional title										
None	–	–	–	–	1	–	–	–	–	1
Primary	−0.211	0.306	0.472	0.492	0.81(0.44,1.48)	0.593	0.175	11.479	0.001	1.81(1.28,2.55)
Intermediate and above	−0.743	.397	3.504	0.061	0.48(0.22,1.04)	0.200	0.194	1.059	0.303	1.22(0.84,1.79)
Work units	–	–	–	–	1	–	–	–	–	1
Mining unit	−1.169	0.289	16.397	< 0.001	0.31(0.18,0.55)	0.347	0.154	5.057	0.025	1.42(1.05,1.92)
Beneficiation unit	−1.383	0.319	18.762	< 0.001	0.25(0.13,0.47)	−0.324	0.185	3.049	0.081	0.72(0.50,1.04)
Smelting unit	–	–	–	–	1	–	–	–	–	1
Job burnout										
Zero burnout	−23.906	< 0.001	–	–	< 0.001	−1.526	0.351	18.885	< 0.001	0.22(0.11,0.43)
Mild burnout	−1.437	0.377	14.538	< 0.001	0.24(0.11,0.50)	−0.809	0.328	6.087	0.014	0.45(0.23,0.85)
Moderate burnout	−1.501	0.368	16.642	< 0.001	0.22(0.11,0.46)	−0.460	0.322	2.032	0.154	0.63(0.34,1.19)
High burnout	–	–	–	–	1	–	–	–	–	1

## Discussion

Given the fast pace of modern life, people work and live under considerable pressure, and the phenomenon of job burnout has appeared in various fields [38]. A series of adverse effects such as fatigue, anxiety, depression, and decline in work ability are also becoming serious. Job burnout has become a key topic in the study of health psychology, and more scholars at home and abroad have studied it [39]. Research shows that the annual economic impact of productivity loss in 2015 was estimated to be a $153.8 million due to psychological distress in the Australian coal industry [40]. In Swedish studies of the working population, the prevalence of burnout has been shown to vary between 6 and 18%, and the public in Sweden sees burnout as a serious public health issue [3]. After conducting a national survey, Shanafelt reported that almost half of American physicians experience some degree of burnout [41]. Another study found that the detection rate of job burnout was 90% in front-line coal miners, with 39.8% experiencing mild burnout, 43.8% with moderate burnout, and 6.4% with high burnout levels [42].

In this survey, we found that there were 1014 miners with different levels of burnout among 1254 copper-nickel miners, accounting for 80.86% of the total: 432 experienced mild burnout (34.45%), 516 had moderate burnout (41.15%), and 66 had high burnout (5.26%). The results suggest that the phenomenon of occupational burnout among copper-nickel miners is serious.

Coal mining is a male-dominated industry. In this survey, we found male burnout was higher than female burnout. Men are the main gender component of copper-nickel miners. Due to the gender roles assigned by their specific society, men rarely discuss the difficulties they face and they lack the means to relieve stress.In addition, male are stigmatized for recognizing and helping to seek mental illness [43]. A long-term fixed shift system and lack of communication with family and friends make it difficult to even acknowledge burnout, and the degree of burnout thus increases over time. According to our results, miners 35–40 years old experience a higher level of job burnout. As they age, miners are increasingly familiar with the technological operation process, but their work is monotonous and repetitive, with little room for promotion and a failure to work under conditions they consider ideal. This reduces their sense of achievement and makes them prone to frustration, leading to less occupational morale and even negative behavior. This, in turn, exacerbates the occupational burnout they experience. Miners with a high-school education have the strongest sense of job burnout, with different recognition and cognitive abilities regarding occupationally harmful factors during the work process. Continuous repetition and intensive work make miners in this group prone to acrimony, increasing the level of burnout. For such workers to improve their position with promotions, they often require more academic qualifications. Thus, opportunities for promotion are relatively few, aggravating job burnout [43]. Moreover, miners with the lowest incomes have the strongest job burnout. As incomes increase, the level of burnout tends to decrease. The amount of remuneration directly reflects the form and mode of labor value. Miners face high labor intensity, and the working environment is harsh. They come into contact with many harmful occupational factors, increasing the potential risk of occupational injury. When high-intensity physical labor does not match the labor return it receives, burnout results. Combined with low monthly income, it is difficult to guarantee a decent quality of life. Therefore, copper-nickel mines bear a heavy mental burden, which increases their level of burnout [44].

When comparing the beneficiation and smelting units, the burnout level of miners in smelting is relatively high, whereas the working ability of miners involved in beneficiation is relatively high. The reasons for this are related to differences in the production process, operation process, and organizational structures. The survey found that the extent to which the mechanical equipment is automated with beneficiation is relatively high. Its labor intensity is lower than that of the mining unit and smelting unit, and the attention of management is higher than that of the other two units. Smelting is more intensive than other jobs, and its tasks are more complicated. Occupational harm (from high temperatures, chemical substances, irritant gases, etc.) is also more common during the production process of smelting than with other units [45].

By comparing the scores of miners’ working ability under different levels of burnout, it was found that with an increase in burnout level, the scores decreased for working ability. Partial correlation analysis showed that job burnout was negatively correlated with work ability. Multinomial logistic regression analyses showed that the education level, professional title, work units and job burnout level have a comprehensive impact on the working ability of copper-nickel miners. The reduced level of education, primary title, smelting unit, and the increase in job burnout are risk factors for the reduction of working ability. The results all showed that job burnout had a negative impact on the working ability of miners, the more intense the job burnout is, the lower the job ability, and reducing job burnout can improve the work ability of copper and nickel miners.

The survey found that, in general, serious job burnout is experienced by miners, and that the relevant departments should take necessary measures to alleviate the level of job burnout to improve the physical and mental health and working ability of miners. First, to create a good working environment, a reasonable management policy and a wage and welfare system is needed, to create a relaxed, harmonious, and positive cultural atmosphere that is conducive to preventing burnout. Reasonable allocation of human resources, facilitated personal promotion, improved personal accomplishment, tasks that are arranged rationally, and an effective occupational exposure prevention system are effective ways to prevent burnout. In addition, the miners themselves should strengthen their professional knowledge and morale. They should continuously improve their own professional and technical level, enhance their individual coping ability, strengthen their mental health maintenance and personality training, and actively respond to stress. Miners should also seek to improve their mental health by seeking the help of a psychologist if necessary.

We recognize that there are limitations associated with our study. The data analysed in this study reflects self-report data collected from Xinjiang coal miners. There may be some questions that involve recall bias. It is not known if the results can be extrapolated to other regions in China, or to other countries or industries. At present, the mechanism of job burnout is unclear. In addition, intervention research on job burnout needs to be carried out.

## Conclusion

Our study found that, owing to their specific working environment, copper and nickel miners experience serious burnout. The burnout level differed depending on sex, age, education level, monthly income, and type of work. Further, with more pronounced job burnout, the job ability suffered. Thus, reducing burnout can improve the working ability of miners. The relevant departments should adopt the necessary measures to alleviate the level of job burnout in miners to improve their working ability.

## Data Availability

The datasets used and analyzed during the current study are available from the corresponding author on reasonable request.

## Abbreviations

CMBI: Chinese Maslach Burnout Inventory
*M*: median
*Q*: quartile
WAI: Work Ability Index Questionnaire

## References

[1] Freudenberger HJ (1975). The staff burn-out syndrome in alternative institutions. Psychotheray Theory Res Pract.

[2] Maslach C, Jackson SE (1981). The measurement of experienced burnout. J Organ Behav.

[3] Aronsson G, Theorell T, Grape T (2017). A systematic review including meta-analysis of work environment and burnout symptoms. BMC Public Health.

[4] Shanafelt TD, Noseworthy JH (2017). Executive leadership and physician well-being: nine organizational strategies to promote engagement and reduce burnout. Mayo Clin Proc.

[5] Huang XF (2008). Status and countermeasures of job burnout of nursing staff in a three-level comprehensive hospital in Shanghai.

[6] Li N (2016). Status causes and countermeasures of job burnout of staff in Chongqing street town office.

[7] Baka L (2015). Does job burnout mediate negative effects of job demands on mental and physical health in a group of teachers? Testing the energetic process of job demands-resources model. Int J Occup Med Environ Health.

[8] Lasalvia A, Bonetto C, Bertani M (2009). Influence of perceived organisational factors on job burnout: survey of community mental health staff. Br J Psychiatry.

[9] Choy HB, Wong MC (2017). Occupational stress and burnout among Hong Kong dentists. Hong Kong Med J.

[10] Zhang Y, Feng X (2011). The relationship between job satisfaction, burnout, and turnover intention among physicians from urban state-owned medical institutions in Hubei, China: A cross-sectional study. BMC Health Serv Res.

[11] Lin QH, Jiang CQ, Lam TH (2013). The relationship between occupational stress, burnout, and turnover intention among managerial staff from a Sino-Japanese joint venture in Guangzhou, China. J Occup Health.

[12] Temam S, Billaudeau N, Vercambre MN (2019). Burnout symptomatology and social support at work independent of the private sphere: a population-based study of french teachers. Int Arch Occup Environ Health.

[13] Li YR, Lu HL, Liu Y (2015). Research progress on occupational burnout of medical staff. Mod Prevent Med.

[14] Vinnikov D, Dushpanova A, Kodasbaev A (2019). Occupational burnout and lifestyle in Kazakhstan cardiologists. Arch Public Health.

[15] Ahola K, Honkonen T, Pirkola S (2006). Alcohol dependence in relation to burnout among the finnish working population. Addiction..

[16] Ahola K, Pulkki-Raback L, Kouvonen A (2012). Burnout and behavior-related health risk factors: results from the population-based Finnish health 2000 study. J Occup Environ Med.

[17] Yao SM, Yu HM, Ai YM (2015). Job-related burnout and the relationship to quality of life among chinese medical college staff. Arch Environ Occup Health.

[18] Njim T, Mbanga CM, Tindong M (2019). Burnout as a correlate of depression among medical students in Cameroon: a cross-sectional study. BMJ Open.

[19] Peterson U, Demerouti E, Bergstrom G (2008). Burnout and physical and mental health among Swedish health care workers. J Adv Nurs.

[20] Verweij H, Frank MM, Madelon LM (2017). The contribution of work characteristics, home characteristics and gender to burnout in medical residents. Adv Health Sci Educ Theory Pract.

[21] Talih F, Warakian R, Ajaltouni J (2016). Correlates of depression and burnout among residents in a Lebanese academic medical center: a cross-sectional study. Acad Psychiatry.

[22] Dewa CS, Loong D, Bonato S (2014). How does burnout affect physician productivity? A systematic literature review. BMC Health Serv Res.

[23] Maslach C, Schaufeli WB, Leiter MP (2001). Job burnout. Annu Rev Psychol.

[24] Wu FY (2018). The current situation of bank staff's job burnout and its impact on health.

[25] Elmariah H, Thomas S, Boggan JC (2017). The burden of burnout. Am J Med Qual.

[26] Goh J, Pfeffer J, Zenios SA (2016). The relationship between workplace stressors and mortality and health costs in the United States. Manag Sci.

[27] Wilke C, Ashton P, Elis T (2015). Analysis of work ability and work-related physical activity of employees in a medium-sized business. BMC Res Notes.

[28] Maslach C, Leiter MP (2016). Understanding the burnout experience: recent research and its implications for psychiatry. World Psychiatry.

[29] Zhao Z, Zhao J, Han M (2015). Status of job burnout and work ability in college teachers. Ind Health Occup Dis..

[30] Schouteten R (2017). Predicting absenteeism: screening for work ability or burnout. Occup Med (Lond).

[31] Ge H (2018). Occupational epidemiological survey of copper-nickel miners and related research on the status of unsafe behavior.

[32] Szigeti R, Balazs N, Bikfalvi R (2017). Burnout and depressive symptoms in teachers: factor structure and construct validity of the maslach burnout inventory-educators survey among elementary and secondary school teachers in Hungary. Stress Health.

[33] Liu N (2013). Study on the relationship between job characteristics, job burnout and team performance.

[34] Li YX, Wu MZ (2005). Research on the structure of job burnout. Psychol Sci.

[35] Li YX, Zhang K, Zhao GX (2005). Analysis of the confirmatory factors of job burnout structure. Psychol Explor.

[36] Li FY, Liu JW, Lian YL (2009). Reliability and validity analysis of mental workers' burnout measurement tools. Ind Hyg Occup Dis.

[37] Ma LJ, Wei Z, Jin TZ (2000). Reliability and validity of the Chinese version of the work ability index questionnaire. Lab Med.

[38] Li HM, Ding W, Zhang J (2018). Investigation on mental health status and burnout level of vehicle drivers in wild oilfields. Xinjiang Med Univ.

[39] Rotenstein LS, Torre M, Ramos MA (2018). Prevalence of burnout among physicians: a systematic review. Jama..

[40] Ling R, Kelly B, Considine R, Tynan R, Searles A, Doran CM (2016). The economic impact of psychological distress in the Australian coal mining industry. J Occup Environ Med.

[41] Shanafelt TD, Boone S, Tan L (2012). Burnout and satisfaction with work-life balance among us physicians relative to the general us population. Arch Intern Med.

[42] He DS (2017). Epidemiological investigation and related factors analysis of occupational burnout and musculoskeletal disorders among coal miners in Xinjiang.

[43] Tynan RJ, Considine R, Rich JL, Skehan J, Wiggers J, Lewin TJ (2016). Help-seeking for mental health problems by employees in the Australian mining industry. BMC Health Serv Res.

[44] Liu JJ, Xue YF, Zhang YZ (2017). A study on mental health and job burnout of occupational groups in Xinjiang. Ind Health Occup Dis.

[45] Li Y, Sun XM, Ge H (2019). The status of occupational stress and its influence the quality of life of copper-nickel miners in Xinjiang, China. Int J Environ Res Public Health.

